# No association between hand and foot temperature responses during local cold stress and rewarming

**DOI:** 10.1007/s00421-017-3601-5

**Published:** 2017-04-18

**Authors:** Lena Norrbrand, Roger Kölegård, Michail E. Keramidas, Igor B. Mekjavic, Ola Eiken

**Affiliations:** 1 Department of Environmental Physiology, School of Technology and Health, KTH Royal Institute of Technology, Berzelius väg 13, SE-171 65 Solna, Sweden; 2 Department of Automation, Biocybernetics and Robotics, Jozef Stefan Institute, Jamova 39, 1000 Ljubljana, Slovenia; 3 Department of Biomedical Physiology and Kinesiology, Simon Fraser University, Burnaby, BC Canada

**Keywords:** CIVD, Finger, Toe, Water immersion, Rewarming, Local cold injury, Cold tolerance

## Abstract

**Purpose:**

The purpose was to examine whether associations exist between temperature responses in the fingers vs. toes and hand vs. foot during local cold-water immersion and rewarming phases.

**Methods:**

Seventy healthy subjects (58 males, 12 females) immersed their right hand or right foot, respectively, in 8 °C water for 30 min (CWI phase), followed by a 15-min spontaneous rewarming (RW) in 25 °C air temperature.

**Results:**

Temperature was lower in the toes than the fingers during the baseline phase (27.8 ± 3.0 vs. 33.9 ± 2.5 °C, *p* < 0.001), parts of the CWI phase (min 20–30: 8.8 ± 0.7 vs. 9.7 ± 1.4 °C, *p* < 0.001), and during the RW phase (peak temperature: 22.5 ± 5.1 vs. 32.7 ± 3.6 °C, *p* < 0.001). Cold-induced vasodilatation (CIVD) was more common in the fingers than in the toes (*p* < 0.001). Within the first 10 min of CWI, 61% of the subjects exhibited a CIVD response in the fingers, while only 6% of the subjects had a CIVD response in the toes. There was a large variability of temperature responses both within and between extremities, and there was a weak correlation between finger- and toe temperature both during the CWI (*r* = 0.21, *p* = 0.08) and the RW phases (*r* = 0.26, *p* = 0.03).

**Conclusions:**

Results suggest that there is generally a lower temperature in the toes than the fingers after a short time of local cold exposure and that the thermal responses of the fingers/hands are not readily transferable to the toes/foot.

## Introduction

Prolonged exposure to low ambient temperatures may induce cold injuries, particularly in the hands and feet; the feet appear to be more vulnerable than the hands (DeGroot et al. 2003; Juopperi et al. 2002). Considering the amount of people exposed to harsh, or even extreme, weather conditions in a rather elective manner, either for occupational or recreational reasons (e.g., military personnel and mountaineers), the establishment and evaluation of a sensitive prediction tool constitute an essential step for the prevention of cold injuries.

Based on the premise that the cold-induced vasodilatation (CIVD) response, a “paradoxical” rise in the digits circulation shortly upon exposure to cold (Lewis 1930), might serve as a cryoprotective mechanism (Wilson and Goldman 1970; Mathew et al. 1973, 1974), it has been suggested that the temperature reaction of the digits in one limb (Van der Struijs et al. 2008), or even one digit (Daanen and van der Struijs 2005), to a local cold stimulus might reflect the overall susceptibility to cold injury, regardless of the testing region. Thus, Daanen and van der Struijs (2005) have advocated that the employment of a short-term cold-provocation test in one finger could be used as a means to stratify individuals into groups possessing low and high risks of cold injury. This notion has since been challenged by Cheung and Mekjavic (2007) who showed that, in a small group of healthy males, the temperature responses during cold stress are not homogenous across, nor between, the hand and foot, and therefore, any generalization of the thermal response stemming from a single region seems unwarranted (Cheung and Mekjavic 2007). This conclusion is also in line with that of Chen et al. (1996).

Not only the temperature reactions during local cooling, but also the thermal responses during a period of spontaneous (passive) rewarming ensuing a local cold stimulus have been considered as an approach to detect the sensitivity to cold (Davey et al. 2013; Eglin et al. 2013) and to indicate susceptibility to cold injury (Ahle et al. 1990; Brändström et al. 2008; Ruijs et al. 2008). Brändström et al. (2008) have argued that the rewarming response of the hand following a 10-min local cooling can be generalized to all extremities predicting the risk of local cold injury, and hence can be used, in a military setting, as a tool to select individuals for redeployment to warmer assignments. Yet, although there are indications that the rewarming responses of the hand may differ from those of the foot (cf. Morrison et al. 2015; Rissanen and Rintamäki 2009), to our knowledge, no study has hitherto systematically examined in a large group of individuals whether the rewarming response of the hand is transferable to the foot. In the aforementioned studies by Chen et al. (1996) and Cheung and Mekjavic (2007), small groups of individuals were investigated (*n* = 8 and *n* = 10, respectively) and only during the cooling phase.

Accordingly, the purpose of the present study was to map the temperature responses of all exposed segments of the hand and foot during and after local cold stress in a large cohort of young individuals and to examine whether the responses of one limb would be transferable to the other limb. Based on results in previous studies (Chen et al. 1996; Cheung and Mekjavic 2007; Morrison et al. 2015; O’Brien 2005; Rissanen and Rintamäki 2009), we hypothesized that: (1) the foot would reach lower temperatures than the hand during both the cold-water immersion and rewarming phases and that (2) the temperature responses of the hand would not be associated with those of the foot neither during nor after the local cold stress.

## Methods

### Subjects

Fifty-eight male and twelve female cadets of the Swedish Armed Forces participated in the study (*n* = 70, age: 23.2 ± 2.4 years, height: 1.80 ± 0.07 m, weight: 78.8 ± 9.9 kg, BMI: 24.4 ± 2.5 kg m^−2^). Subjects were healthy, had no history of any cold injury, and no previous experience with cold-exposure experiments. They were informed in detail about the experimental procedures and gave their written consent. They were instructed not to engage in any strenuous activity, to refrain from consuming any caffeinated product for a day before, and not to use tobacco within 6–8 h prior to the test. The experimental protocol was approved by the Humans Ethics Committee of Stockholm and conformed to the Declaration of Helsinki.

### Experimental protocol

Subjects performed, in a counterbalanced order and separated by a ~15-min interval, two cold-water immersion tests: once they immersed the right hand, and on the other occasion, the right foot. Subjects were dressed in T-shirt and shorts (they also had socks during the hand cold test), and remained in a sitting position throughout each test. Prior to the start of the test, subjects were accustomed to the conditions of the laboratory for ~20 min. The mean temperature and relative humidity in the laboratory were 25.0 ± 0.5 °C and 36 ± 9%, respectively. All tests were performed between November and February.

Each test commenced with a 5-min baseline phase, during which the subject rested with the test limb dry in room temperature on a support (baseline phase). After this, the limb was covered with a thin plastic bag (thickness of 0.025 mm), sealed with air-permeable tape to the skin (~10 cm above the wrist and ankle, respectively), whereafter it was immersed in warm water (35 °C) for 5 min (WWI phase). The hand was immersed up to the ulnar and radial styloids, and the foot up to the tibial and fibular styloids. Subsequently, the limb was removed from the warm-water tank, and placed without the plastic bag on the limb support for ~1 min, during which infrared-thermal images were obtained (see below for details). Thereafter, the hand/foot was covered with a new plastic bag and immersed in a tank containing cold water (8 °C) for 30 min (CWI phase). The temperature of the water was maintained by means of a cooling system (Cylinda, Elektroscandia Sverige AB, Sollentuna, Sweden), and a pump continuously stirred the water. After completion of the CWI phase, the limb was removed from the water, dried with a towel, if necessary, and a 15-min spontaneous rewarming (RW) phase ensued, while the limb was resting as in the baseline phase. Throughout each test, the subject was instructed to keep the contralateral (non-immersed) limb immobile on the support.

### Instrumentation

#### Thermocouple probes

During each test, digit skin temperatures of the immersed limb were measured with copper-constantan (T-type) thermocouple probes (Physitemp Instruments Inc., Clifton, NJ, USA), attached to the middle of the volar side of the distal phalanx of each digit. An additional thermocouple probe was placed at the center of the dorsal metacarpal and metatarsal region of the hand (H-MC) and foot (F-MT), respectively. The primary insulation of the thermocouples was polytetrafluoroethylene; the non-insulated welded junction of each thermocouple was attached directly to the skin with thin air-permeable tape (Tegaderm, 3M, Healthcare, St. Paul, MN, USA). Skin temperatures were sampled every second with an NI USB-6215 (National Instruments, Austin, Texas, USA) data acquisition board, operated with the TestPoint software (TestPoint v7^®^, Norton, Massachusetts, USA). Following a manual check of the raw data, a custom-made computer program (TestPoint) was used to calculate the average temperature (*T*_avg_) of each digit during every phase, as well as the minimum (*T*_min_) and maximum (*T*_max_) temperatures reached during the CWI and RW phases. The same program was also used to detect any digit CIVD response, defined as a local skin-temperature wave (*N*) in terms of >1 °C increase lasting for a minimum duration of 3 min. In case of a CIVD response, the following parameters were determined: (1) the temperature amplitude (Δ*T*), which was the difference between the lowest temperature recorded just before the CIVD and the highest temperature reached during the CIVD and (2) the onset time (*t*_onset_) of the first CIVD response.

#### Infrared thermography

During the dry phases of the experiment, i.e., at the end of the baseline, immediately after the WWI, and during the RW phase (at minutes 1, 5, 10 and 15), skin temperatures on the volar and dorsal side of the hand/foot, that was to be or had been immersed, were recorded with an infrared camera (FLIR T365, FLIR Systems AB, Danderyd, Sweden), which was calibrated automatically. Infrared images of the contralateral, non-immersed limb were also taken during the baseline, after the WWI phase, and at the first and fifth minute of the CWI phase. The field of the camera’s view was 25° × 19°, the spatial resolution was 1.36 mrad, the spectral range 7.5–13 µm, and the infrared detector resolution was 320 × 240 pixels. The distance between the camera and the extremity was ~60 cm. The thermal images were analyzed using the ThermaCam Researcher PRO 2.10 software (FLIR Systems AB, Danderyd, Sweden). In 69 of the subjects (for technical reasons, the data from one subject could not be analyzed in the foot that had been immersed), *T*_avg_ of the immersed hand was determined for the following anatomical areas: (1) the volar and the dorsal side of the distal phalanx of each finger; (2) the total palm; and (3) dorsal metacarpal area. In the same subjects, *T*_avg_ of the immersed foot was determined for the following areas: (1) the volar and the dorsal side of the distal phalanx of each toe; (2) the total sole; and (3) dorsal metatarsal area. In all 70 subjects, *T*_avg_ of the contralateral, non-immersed hand, and foot was determined for the following anatomical areas: (1) the volar side of the distal phalanx of finger II; (2) the total palm; (3) the volar side of the distal phalanx of toe II; and (4) the total sole.

#### Tympanic temperature

During the baseline and RW phases, the tympanic temperature (*T*_tympanic_) was measured using an infrared thermometer (ThermoScan IRT 6012, Braun, Kronberg, Germany). Two consecutive measurements were obtained each time, and the higher of the two values was used for subsequent analysis.

#### Haemodynamic variables

Heart rate (HR), systolic (SAP), and diastolic (DAP) arterial pressures were measured at 5-min intervals using an automated oscillometric sphygmomanometer (Omron M6, Kyoto, Japan) with the cuff positioned around the mid portion of the left upper arm.

#### Psychometric response scales

During the baseline, WWI, CWI (at minutes 1, 2, 3, 4, 5, and every 5 min thereafter) and RW phases, subjects were asked to provide ratings of thermal sensation (from 1-cold to 7-hot), thermal comfort (from 1-comfortable to 4-very uncomfortable), and local pain (from 0-no pain to 10-unbearable pain). All scales were explained to the subjects prior to each test.

### Statistical analysis

Statistical analyses were performed using Statistica 7 (StatSoft, Inc., Tulsa, Oklahoma, USA). All variables are presented as mean ± SD, unless otherwise indicated. A two-way general linear model repeated measures ANOVA was used to evaluate the thermal (limb × region), and haemodynamic (limb × phase) responses within and between the two tests. When ANOVA revealed a significant *F* ratio for interaction and/or main effect, pairwise comparisons were performed with Tukey HSD post hoc test. Pearson product-moment correlation was used to evaluate the relation between selected physiological variables. Magnitudes of correlations were interpreted qualitatively using Evans guidelines (Evans 1996): *r* = 0.00–0.19: very weak, 0.20–0.39: weak, 0.40–0.59: moderate, 0.60–0.79: strong, 0.80–1.0: very strong. Differences in *N*, thermal comfort, thermal sensation, and local pain were evaluated with a Wilcoxon matched pairs nonparametric test. The alpha level of significance was set a priori at 0.05.

## Results

### Temperature responses

#### Immersed limb

##### Baseline and WWI phases

During both phases, the toes (baseline: 27.8 ± 3.0 °C, WWI: 33.0 ± 1.6 °C) and F-MT (baseline: 32.2 ± 1.3 °C, WWI: 34.4 ± 0.8 °C) were colder (*p* < 0.001) than the fingers (baseline: 33.9 ± 2.5 °C, WWI: 35.6 ± 1.0 °C) and H-MC (baseline: 33.2 ± 1.4 °C, WWI: 35.2 ± 0.8 °C), respectively.

##### CWI phase

**Fig. 1:**
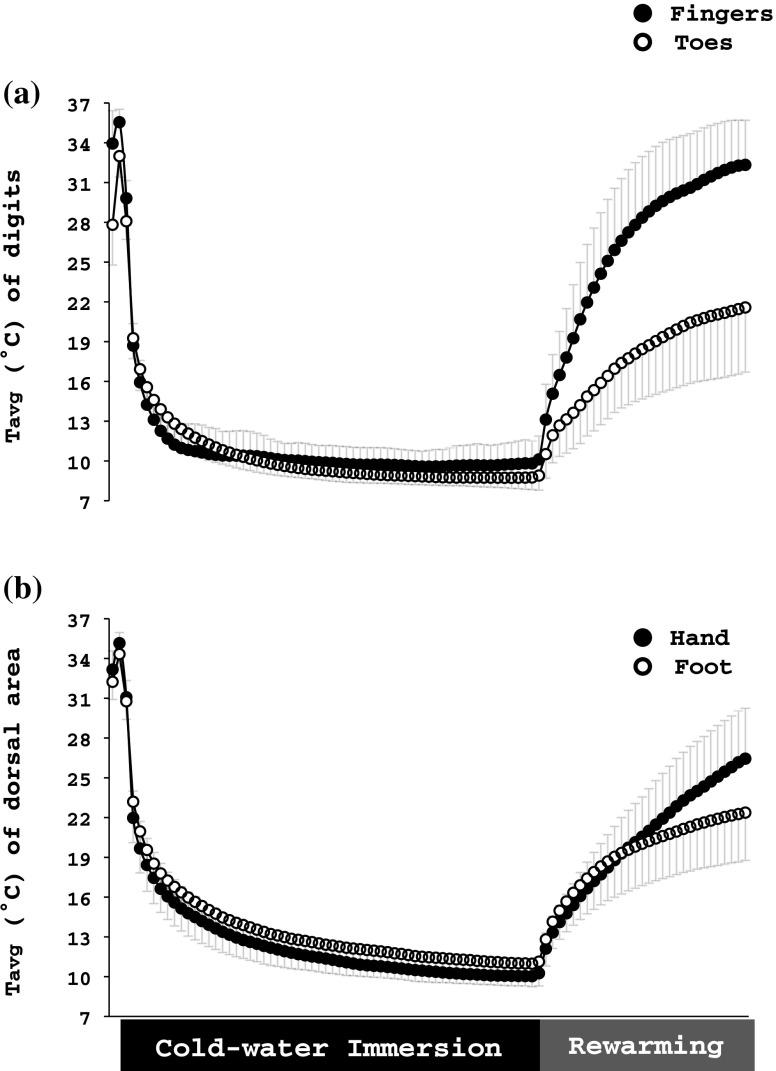
Average temperature (*T*avg) of (**a**) the distal phalanx of the volar side of all fingers and toes and (**b**) the dorsal metacarpal and metatarsal region of the right hand and foot, respectively, obtained during the cold-water immersion test. Values are mean ± SD. Data are averaged every 30 s

Upon immersion to the cold water, all regions of the hand cooled faster than those of the foot (hand: −5.5 ± 0.4°C·min^−1^; foot:−4.6 ± 0.7°C·min^−1^; *p* < 0.001; Fig. 1). However, after the third minute until the end of the CWI phase, the toes tended to be colder than the fingers (*p* = 0.07; Table 1); especially, during the last 10 min (*p* < 0.001). Conversely, H-MC (12.3 ± 1.0 °C) was colder (*p* < 0.001) than F-MT (13.4 ± 2.1 °C). Between the third minute until the end of the CWI phase, finger I and II were warmer than the other fingers (*p* ≤ 0.002), and toe III, IV, and V were warmer than toe I and II (*p* < 0.001, Table 1).

**Table 1:** Overall average temperature (*T*_avg_), average temperature from the 20th to 30th min (*T*_avg_^20–30^), minimum temperature (*T*_min_), maximum temperature (*T*_max_), number of local skin temperature waves (*N*), temperature amplitude of the wave (Δ*T*), and onset time of the first wave (*t*_onset_) on the volar side of the distal phalanx of each finger and toe obtained from thermocouples during minutes 3–30 of the hand and foot cold-water immersion test

	Hand cold-water immersion test						Foot cold-water immersion test					
	Finger						Toe					
	I	II	III	IV	V	Average	I	II	III	IV	V	Average
*T*_avg_ (°C)	10.9 ± 1.5	10.8 ± 1.5	10.6 ± 1.3	10.3 ± 1.4	10.1 ± 1.1	10.6 ± 1.4	9.7 ± 0.8	9.9 ± 1.3	10.7 ± 1.0	10.3 ± 0.9	10.7 ± 0.9	10.3 ± 1.1
*T*_avg_^20–30^ (°C)	10.1 ± 1.6	10.0 ± 1.5	9.7 ± 1.7	9.5 ± 1.8	9.3 ± 1.2	9.7 ± 1.4	8.5 ± 0.8^c^	8.3 ± 0.9^c^	9.1 ± 0.9^d^	8.8 ± 0.8^c^	9.1 ± 0.8^d^	8.8 ± 0.7^b^
*T*_min_ (°C)	9.0 ± 1.0	9.0 ± 0.8	8.7 ± 0.8	8.6 ± 0.7	8.5 ± 0.7	8.7 ± 0.8	8.1 ± 0.5^c^	8.0 ± 0.6^c^	8.7 ± 0.6^e^	8.5 ± 0.5^e^	8.7 ± 0.4^e^	8.4 ± 0.6^b^
*T*_max_ (°C)	13.4 ± 3.0	12.8 ± 2.9	12.8 ± 2.5	12.4 ± 3.0	12.0 ± 2.4	12.7 ± 2.8	12.1 ± 1.5	13.2 ± 3.1	14.1 ± 2.2	13.1 ± 1.7	13.9 ± 1.9	13.3 ± 2.2
*N*	1(0–3)	1(0–3)	1(0–4)	1(0–4)	1(0–4)	5(0–16)	0(0–2)	0(0–1)	0(0–2)	0(0–2)	0(0–2)	0(0–9)^b^
Δ*T* (°C)^a^	2.7 ± 1.6	2.5 ± 1.5	2.5 ± 1.1	2.2 ± 0.8	2.4 ± 1.3	2.3 ± 0.9	2.1 ± 0.7	2.2 ± 1.0	2.0 ± 0.8	2.0 ± 1.1	2.4 ± 0.8	2.0 ± 0.7
*t*_onset_ (sec)^a^	733 ± 525	624 ± 383	592 ± 391	456 ± 292	416 ± 236	664 ± 362	1079 ± 292	1147 ± 265	1011 ± 323	1049 ± 450	977 ± 238	1051 ± 331^b^

Values are mean ± SD. Values are median (range) for *N*

^a^Comparisons of average Δ*T* and average *t*_onset_ were computed for those subjects, who exhibited at least one CIVD in one toe and one finger (*n* = 25). Results of individual digits are based on uneven numbers of observations

^b^Significant overall difference between fingers and toes

^c^Significantly different from all the fingers

^d^Significantly different from fingers I–IV

^e^Significantly different from fingers I and II

The number of CIVD responses was higher in the fingers than in the toes (*p* < 0.001; Table 1). 43 (61%) subjects exhibited a CIVD response, at least in 1 finger, within the first 10 min of the CWI phase, whereas only 4 subjects (6%) exhibited a CIVD response in the toes during the same time period. All in all, 64 (91%) subjects demonstrated a total of 397 CIVD responses in the fingers, where 30 (43%) subjects exhibited CIVD responses in all fingers. For the toes, 26 (37%) subjects demonstrated a total of 70 CIVD responses, while only 3 (4%) subjects exhibited CIVD responses in all toes. *T*_min_ was lower in the toes than in the fingers (*p* < 0.001; Table 1); there was no difference between the limbs as regards *T*_max_ (*p* = 0.10; Table 1).

##### RW phase

**Fig. 2:**
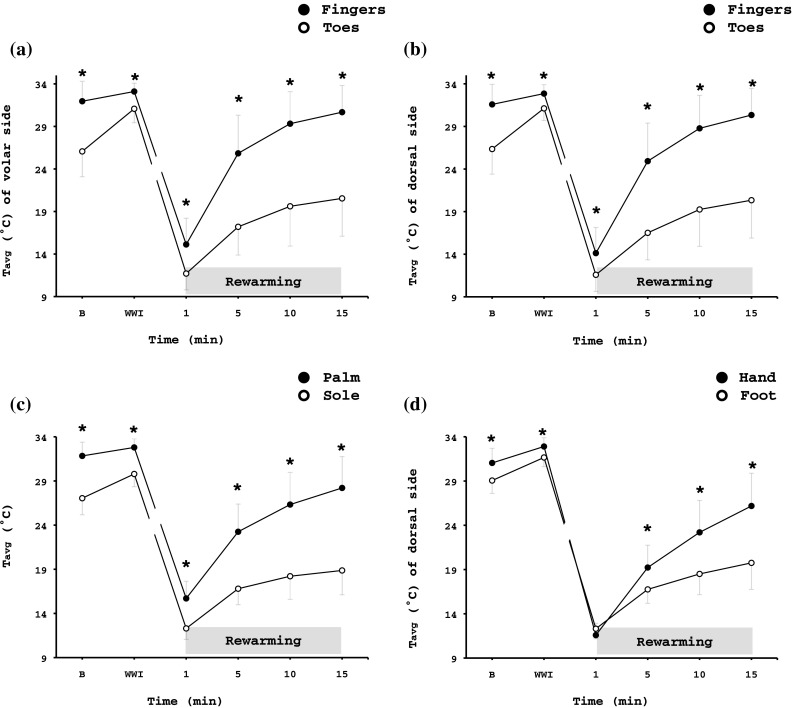
Thermography results showing average temperature (*T*_avg_) of (**a**) the volar and (**b**) the dorsal side of the distal phalanx of the fingers and toes, (**c**) the total area of palm and sole, and (**d**) the total dorsal metacarpal/metatarsal area of the right hand and foot during the spontaneous rewarming phase. Values are mean ± SD. *n* = 69. *Significant difference between hand and foot (*p* < 0.001). *B*: a 5-min baseline phase, *WWI*: a 5-min warm-water immersion phase, *RW*: a 15-min rewarming phase

**Fig. 3:**
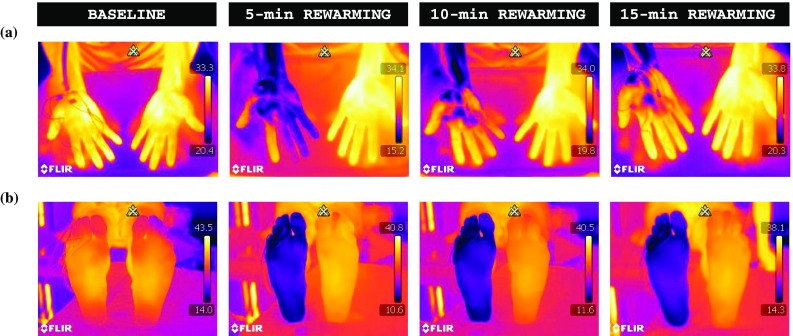
Representative infrared-thermal images from a single subject during the baseline and the 15-min rewarming response of (**a**) the hand and (**b**) the foot. *Color coding* of temperatures are indicated on the right-hand side; the *brighter colors* represent a warmer limb (*yellow*), and the *darker colors* represent a colder limb (*dark purple*). Note (*1*) the rate of rewarming in the hand vs. the foot and (*2*) the distal to proximal rewarming pattern for the fingers

At the onset of the RW phase, *T*_avg_ was higher (*p* < 0.001) in the fingers (9.9 ± 2.0 °C) than in the toes (8.8 ± 1.2 °C, Table 2), but it was lower (*p* < 0.001) in the H-MC (10.1 ± 2.0 °C) than in the F-MT (11.1 ± 1.8 °C). The overall rewarming response of the hand was greater than that of the foot (*p* < 0.001; Table 2; Figs. 2, 3). The fingers and H-MC reached higher *T*max than the toes and F-MT, respectively (Fingers = 32.7 ± 3.6 °C, Toes = 22.5 ± 5.1 °C; H-MC = 27.0 ± 3.9 °C, F-MT = 23.6 ± 3.7 °C; *p* < 0.001). During the RW phase, finger II was warmer than finger IV and V (*p* ≤ 0.003), and toe I was warmer than toe III, IV, and V (*p* < 0.001, Table 2).

**Table 2:** Overall average temperature (*T*_avg_), minimum temperature (*T*_min_), and maximum temperature (*T*_max_) of the palmar side of the distal phalanx of each finger and toe obtained from thermocouples during the 15-min hand and foot rewarming phase

	After cold-water immersion test for hand						After cold-water immersion test for foot					
	Finger						Toe					
	I	II	III	IV	V	Average	I	II	III	IV	V	Average
*T*_avg_ (°C)	26.4 ± 3.6	27.1 ± 3.6	26.6 ± 3.8	26.1 ± 4.2	26.1 ± 4.0	26.5 ± 3.8	18.6 ± 4.0^b^	17.9 ± 4.1^b^	17.7 ± 3.6^b^	17.6 ± 3.6^b^	17.7 ± 3.3^b^	17.9 ± 3.7^a^
*T*_min_ (°C)	10.3 ± 1.9	10.2 ± 1.8	10.0 ± 2.1	9.7 ± 2.3	9.4 ± 2.3	9.9 ± 2.0	8.6 ± 1.0^b^	8.3 ± 1.1^b^	9.1 ± 1.2^c^	9.0 ± 1.2^b^	9.2 ± 1.1^c^	8.8 ± 1.2^a^
*T*_max_ (°C)	32.8 ± 3.1	32.9 ± 3.1	33.0 ± 4.3	32.6 ± 3.9	32.4 ± 3.8	32.7 ± 3.6	23.6 ± 5.5^b^	23.0 ± 5.5^b^	22.4 ± 5.1^b^	21.7 ± 4.8^b^	21.9 ± 4.6^b^	22.5 ± 5.1^a^

Values are mean ± SD

^a^Significant overall difference between hand and foot

^b^Significantly different from all the fingers

^c^Significantly different from fingers I–IV

##### Correlations

**Fig. 4:**
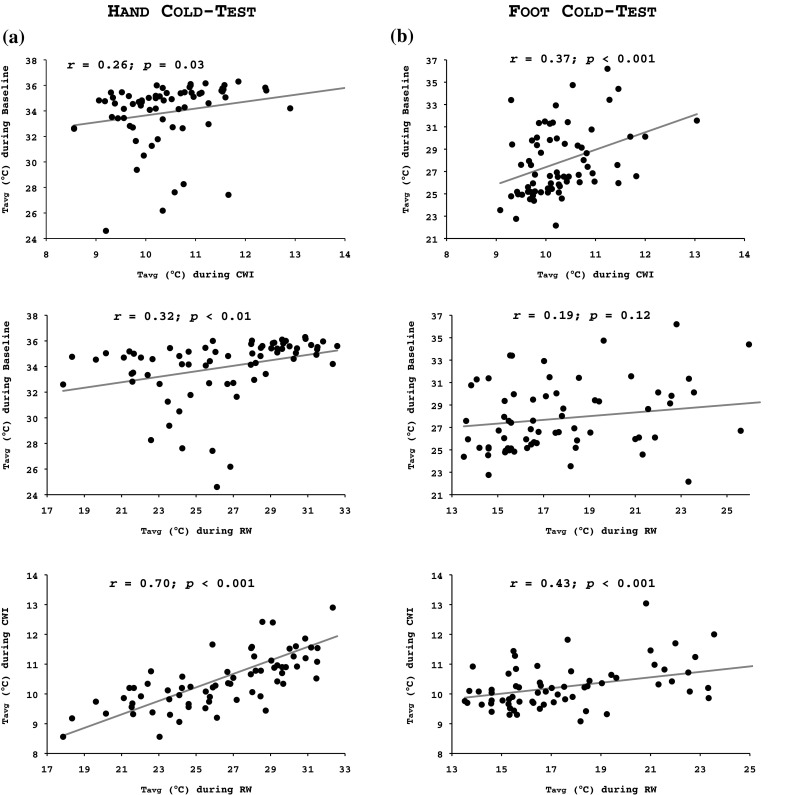
Pearson’s product-moment correlation between the average temperature (*T*_avg_) during the 5-min baseline and the 30-min cold-water immersion phase (*upper graphs*), and the 5-min baseline and the 15-min spontaneous rewarming phase (*middle graphs*), and the 30-min cold-water immersion phase and the 15-min spontaneous rewarming phase (*lower graphs*) in (**a**) all fingers of the right hand and (**b**) all toes of the right foot

A weak correlation was noted between the fingers baseline *T*_avg_ and their *T*_avg_ during the CWI (*r* = 0.26, *p* = 0.03) and the RW (*r* = 0.32, *p* < 0.01) phases (Fig. 4a). The RW *T*_avg_ of the fingers was strongly related to their CWI *T*_avg_ (*r* = 0.70, *p* < 0.001; Fig. 4a). A weak correlation was observed between the toes baseline *T*_avg_ and their CWI *T*_avg_ (*r* = 0.37, *p* < 0.001; Fig. 4b). The RW *T*_avg_ of the toes was moderately correlated to their CWI *T*_avg_ (*r* = 0.43, *p* < 0.001), but not to their baseline *T*_avg_ (*r* = 0.19; *p* = 0.12) (Fig. 4b).

**Fig. 5:**
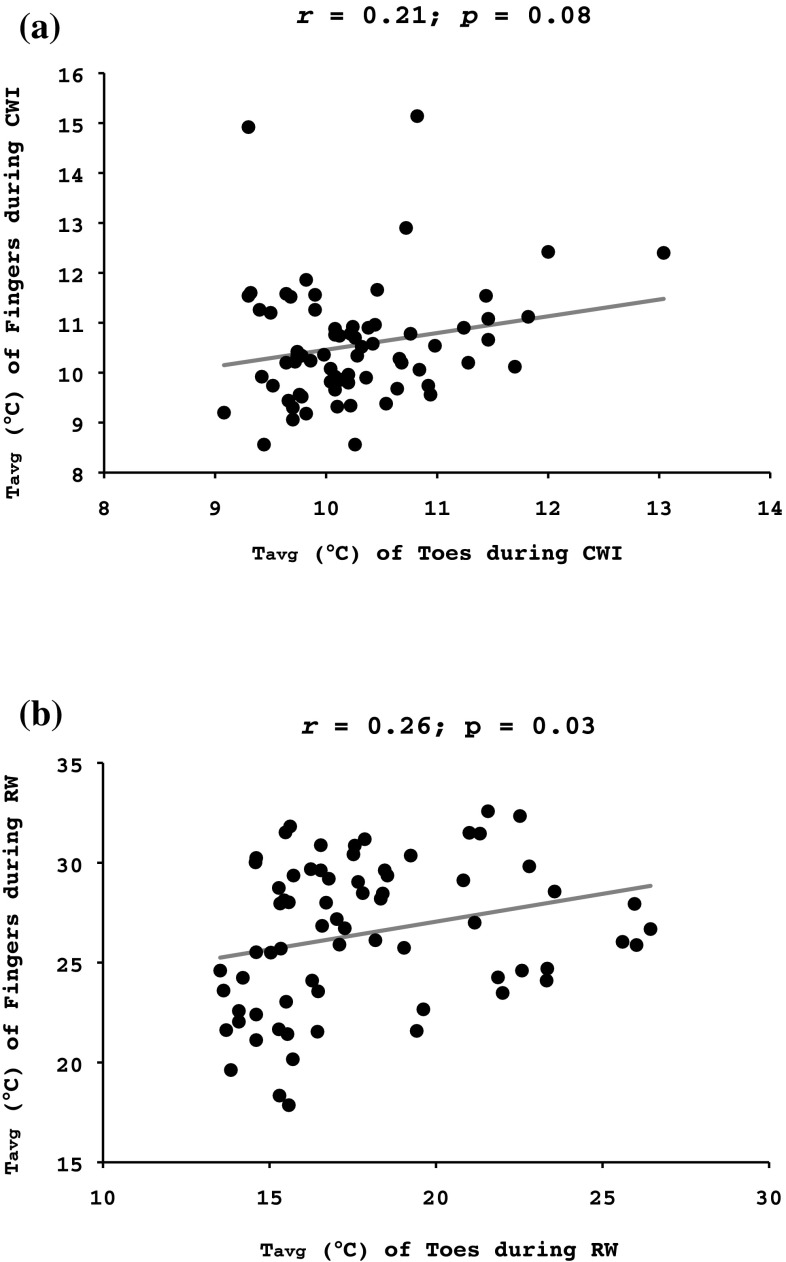
Pearson’s product-moment correlation between the average temperature (*T*_avg_) of all fingers of the right hand and all toes of the right foot during (**a**) the 30-min cold-water immersion phase and (**b**) the 15-min spontaneous rewarming phase

There was a statistical tendency for a weak correlation between the fingers and toes *T*_avg_ during the CWI phase (*r* = 0.21, *p* = 0.08; Fig. 5a). A weak correlation was detected between the RW *T*_avg_ of the digits of the two limbs (*r* = 0.26, *p* = 0.03; Fig. 5b).

#### Non-immersed limb

**Fig. 6:**
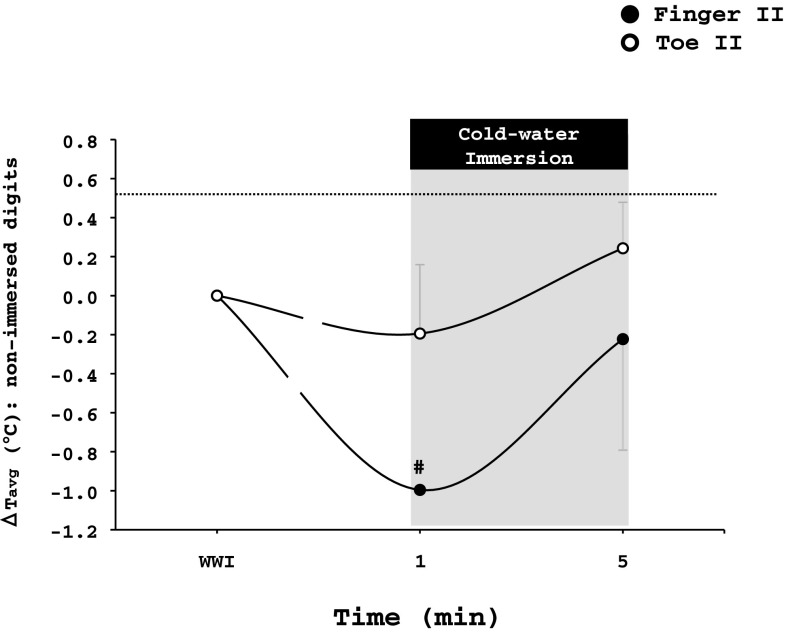
Changes from the warm-water immersion (WWI) phase in the average temperature (*T*_avg_) of the non-immersed finger II and toe II during the hand and foot cold-water immersion test, respectively. Values are mean ± SD. *#* Significant difference from the previous timepoint (*p* ≤ 0.05)

Throughout the tests, the non-immersed hand (finger II: 31.0 ± 3.0 °C, palm: 32.5 ± 1.7 °C) was warmer (*p* < 0.001) than the non-immersed foot (toe II: 26.2 ± 4.3 °C, sole: 27.9 ± 2.6 °C). The cold stimulus did not alter *T*_avg_ of the non-immersed palm or sole (*p* > 0.05). However, upon immersion to cold water, there was a transient drop of ~1 °C in *T*avg of finger II (*p* = 0.01). *T*_avg_ of toe II was also slightly reduced (~0.2 °C), but the drop was not statistically significant (*p* > 0.05; Fig. 6).

#### *T*tympanic

There was no difference in *T*_tympanic_ between the tests (hand test: baseline = 36.5 ± 0.4 °C, RW = 36.5 ± 0.4 °C; foot test: baseline = 36.5 ± 0.4 °C, RW = 36.6 ± 0.4 °C; *p* > 0.05).

### Haemodynamic responses

#### Heart rate

**Fig. 7:**
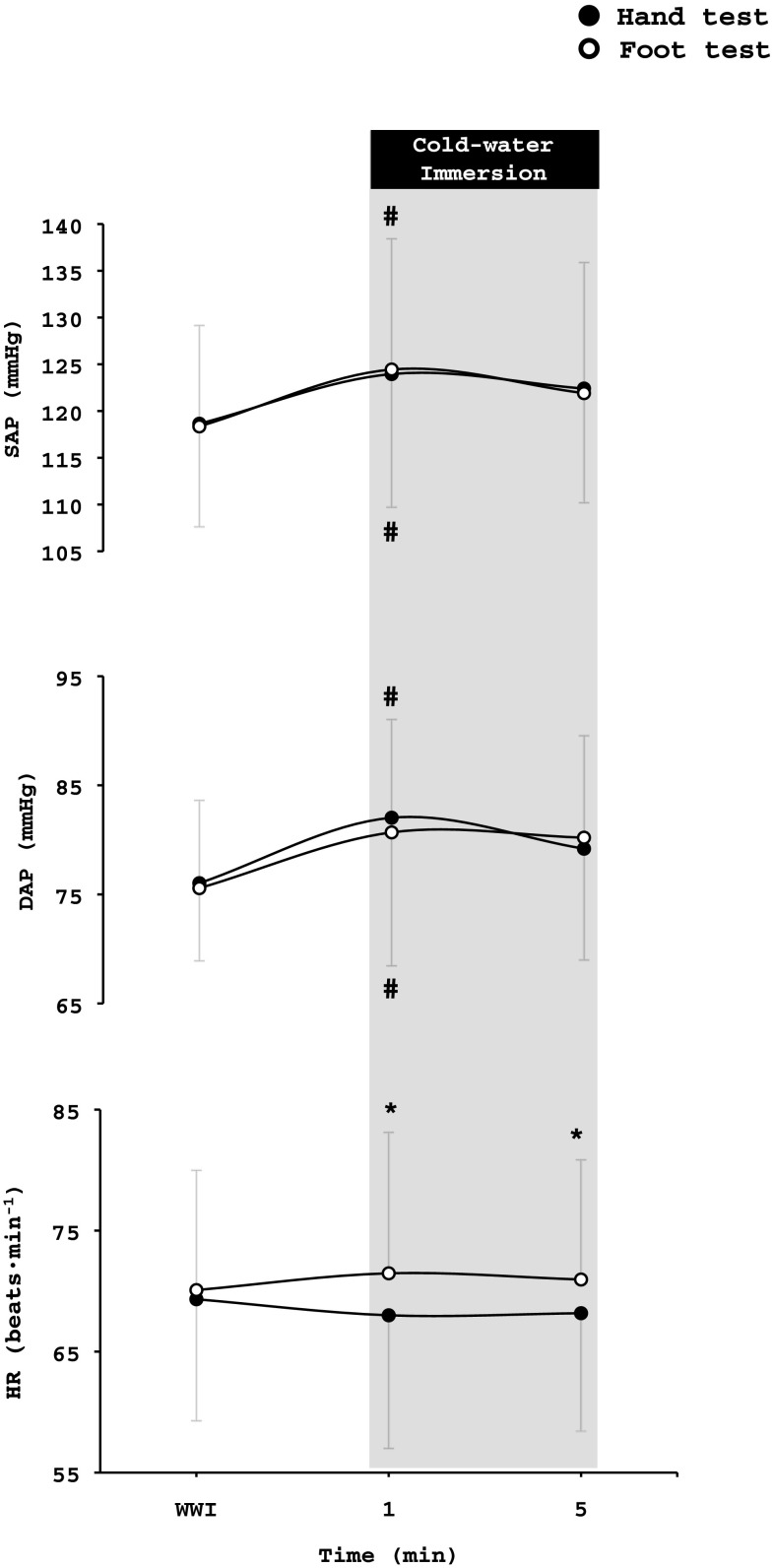
Changes from the warm-water immersion (WWI) phase in systolic (SAP) and diastolic (DAP) arterial pressure, and heart rate (HR) during the hand and foot cold-water immersion test, respectively. Values are mean ± SD. *Significant difference between hand and foot test (*p* < 0.05) ^#^Significant difference in arterial pressure from the previous phase (*p* < 0.05)

Baseline and WWI average values for HR were similar in the hand and foot test, whereas during the CWI and RW phase, HR was higher (*p* < 0.001) during the foot test than the hand test (hand test: baseline = 68 ± 11 beats·min^−1^, WWI = 69 ± 9 beats·min^−1^, CWI = 68 ± 9 beats·min^−1^, RW = 65 ± 9 beats·min^−1^; foot test: baseline = 69 ± 11 beats·min^−1^, WWI = 70 ± 10 beats·min^−1^, CWI = 72 ± 10 beats·min^−1^, RW = 69 ± 10 beats·min^−1^). During the first 5 min of CWI, HR was higher during the foot test than the hand test (*p* < 0.016, Fig. 7).

#### Systolic and diastolic arterial pressure

There were no differences between the hand and foot tests as regards SAP (hand test: baseline = 119 ± 11 mmHg, WWI = 119 ± 11 mmHg, CWI = 122 ± 10 mmHg, RW = 118 ± 9 mmHg; foot test: baseline = 122 ± 11 mmHg, WWI = 118 ± 11 mmHg, CWI = 122 ± 11 mmHg, RW = 119 ± 10 mmHg) or DAP (hand test: baseline = 75 ± 9 mmHg, WWI = 76 ± 7 mmHg, CWI = 79 ± 7 mmHg, RW = 76 ± 7 mmHg; foot test: baseline = 78 ± 8 mmHg, WWI = 76 ± 8 mmHg, CWI = 80 ± 6 mmHg, RW = 78 ± 6 mmHg) responses. During the hand and foot tests, both SAP (*p* < 0.002) and DAP (*p* < 0.002) increased during the first minute of CWI (Fig. 7).

### Psychometric responses

#### Thermal sensation

During both CWI phases, subjects reported similar values of thermal sensation; however, during the RW phase, the foot felt colder than the hand [hand test: 5-min RW = 3 (range 1–7), 10-min RW = 4 (1–6), 15-min RW = 4 (2–7); foot test: 5-min RW = 2 (1–5), 10-min RW = 3 (1–5), 15-min RW = 3.5 (1–6); *p* < 0.001].

#### Thermal comfort

During the last 5 min of CWI and the first 5 min of the RW, subjects felt more uncomfortable in the foot than the hand [hand test: 25-min CWI = 2 (1–4), 30-min CWI = 2 (1–4), 5-min RW = 1.25 (1–4); foot test: 25-min CWI = 3 (1–4), 30-min CWI = 3 (1–4), 5-min RW = 2 (1–4); *p* < 0.001].

#### Pain

During the last 5 min of CWI and the first 5 min of the RW, subjects experienced more pain in the foot than the hand [hand test: 25-min CWI = 2.5 (0–9), 30-min CWI = 2 (0–9), 5-min RW = 0 (0–9); foot test: 25-min CWI = 3 (0–9), 30-min CWI = 3 (0–9), 5-min RW = 1 (0–8); *p* < 0.001].

## Discussion

Based on the presumption that the CIVD response of the digits has a cryoprotective function (Mathew et al. 1973, 1974; Wilson and Goldman 1970) and that an efficient RW response will also protect against cold injury (Francis and Golden 1985), it has been suggested that, in any given individual, the temperature reaction of the digits of one limb, or one digit, during (Daanen and van der Struijs 2005; Van der Struijs et al. 2008) or after (Brändström et al. 2008) a local cold stress is representative of all regions and hence can be used as a means to predict this individual’s general vulnerability to cold injury. Still, a number of reports (Chen et al. 1996; Cheung and Mekjavic 2007; Reynolds et al. 2007) have, by describing the cold-induced thermal reactions in small groups of healthy individuals, indicated that the response is heterogeneous within different regions of a limb, and differ between hand and foot, and accordingly, the response of a specific region is plausibly not generalizable. In the present study, examining the skin temperature reaction of all immersed segments of the hand and foot in a large cohort of normothermic individuals, a weak correlation was observed between the fingers and toes during rewarming and a tendency of correlation between the fingers and toes was found during local cooling; there were no correlations between the hand and the foot neither during cooling nor rewarming. Hence, from a practical viewpoint, current findings confirm and extend previous evidence (Chen et al. 1996; Cheung and Mekjavic 2007; Reynolds et al. 2007) that in a given individual, the local thermal response to cooling is not transferable to other body regions. Considering the weak correlations between finger and toe temperature responses in the present study, including 70 subjects, it is not surprising that no tendencies of such correlations were revealed in the study by Cheung and Mekjavic that included only 10 subjects and investigated only the CWI phase; a power analysis revealed that 58 subjects was needed to exhibit a correlation between finger and toe temperature responses during the present RW. Presumably, in a larger cohort, a statistically significant yet weak correlation between finger and toe temperature responses would be revealed also for the CWI phase. Regardless, such weak correlations have no practical consequence. The sensitivity of the cold-provocation test to predict cold injury still needs to be established (cf. Cheung 2015); yet present results imply that the test’s specificity is not sufficient to justify it being used to, based on the response of a single finger, toe, hand, or foot, identify individuals with a general high risk of developing cold injury.

During the CWI phase, the toes exhibited a more profound drop in *T*_avg_ and less of a CIVD response than did the fingers. After the cessation of the cold stimulus, a more protracted vasoconstriction was also observed in the foot, reflecting an impaired RW response. These findings appear to be in line with those from epidemiological studies (DeGroot et al. 2003; Juopperi et al. 2002) showing that the feet, and particularly the toes, are more vulnerable to cold injury than are the hands/fingers. The mechanisms underlying the heterogeneous thermal response of the two limbs to identical local cold stimuli remains to be established; yet, functional and structural vascular differences should be considered. Thus, presumably as a consequence of a considerably higher hydrostatic pressure loading, in resting humans, precapillary vessels of the distal leg exhibit considerably higher myogenic tone than do the corresponding vessels of the arm (Eiken and Kölegård 2004; Eiken et al. 2014), and blood flow per mass unit is considerably lower in the foot than in the hand (for review, see Taylor et al. 2014).

A number of studies have suggested that the skin-temperature reactions of the digits during local cooling are dependent, at least to some extent, on their pre-immersion temperature (Greenfield et al. 1952; Yamazaki 2010; Keramidas et al. 2014, 2015). However, in the current study, we failed to confirm such a relation, as indicated by the weak correlation in both limbs between *T*_avg_ during the baseline and CWI phases. Likewise, the RW response of the limbs was independent of their thermal status during the baseline phase. Yet, the RW response of the hand and foot was closely associated with its respective response during the CWI phase, a correlation that seemed to be stronger for the hand.

In clinical settings, the assessment of cold sensitivity, assuming to predict vulnerability to cold injury, is commonly based on the skin-temperature response of the limb during a period of spontaneous RW following a 2-min cold-provocation test (Davey et al. 2013; Eglin et al. 2013). Yet, in keeping with the current finding, it has been shown that the magnitude of the rewarming response is largely dependent on the duration of the cooling phase (Wolff and Pochin 1949), during which a fall in the temperature of deeper tissue layers is induced (Barcroft and Edholm 1943). Hence, considering the aforementioned relation between basal temperature and the thermal response to CWI, as well as the slower cooling rate of the foot, that may be due to its greater mass to surface area (cf. Jay and Havenith 2004), present results might imply that a longer cold-provocation than 2 min is warranted when the cold sensitivity of the foot is evaluated, or is compared with that of the hand.

Judging by the HR, but not the arterial pressure, responses, the cold-water immersion of the foot evoked a greater overall sympathetic reaction; a similar response has been reported previously (Amon 2009). Any enhanced sympathetic excitation during the foot cold test might be a result of the greater mass area submersed to cold water in this condition (cf. Sendowski et al. 1997). In this regard, the findings by Larra et al. (2015) showing that a bilateral foot cold-pressor test exaggerated the elevations in HR and the salivary alpha-amylase that were induced by a unilateral hand cold test are also of interest.

During the latter part of the CWI and during the RW, temperature was perceived considerably more uncomfortable and painful in the foot trial than the hand trial. Conceivably, this difference in perceived discomfort/pain reflected concomitant lower temperatures in the toes than the fingers; during this period, skin temperatures were, by contrast, generally higher in the F-MT than the H-MC region. Regardless of the reason, the exaggerated discomfort in the toes during and following a given cold stress is noteworthy considering that cold injuries appear to be more common in the toes than the fingers (DeGroot et al. 2003; Juopperi et al. 2002) and that behavioral alterations are regarded our first line of defense against cold environments (Mekjavic and Eiken 2006). In field conditions, however, it is commonly easier to behaviorally respond to cold discomfort in the hands (add insulation, and rewarming etc) than in the feet.

Upon immersion to cold water, a transient cutaneous vasoconstriction was observed in the non-immersed digits, but not in the other segments of the limbs. Although the mechanisms underlying this short-lasting vasoconstriction are not fully understood, it has been suggested to be mediated either via a reflex response to a noxious stimulus, and/or to be the action of a central mechanism elicited by the fall in blood temperature (Folkow et al. 1963; Kregel et al. 1992; Marshall et al. 1990; Pickering 1932). Notably, in the present study, the “indirect” vasoconstriction in the non-immersed digits was more pronounced in the contralateral finger than in the contralateral toe. The mechanisms underlying this discrepancy need to be further investigated. A blunted neural stimulus for the foot, resulting from a slower initial cooling rate and a smaller absolute drop in skin temperature, might have contributed to the weaker indirect vasoconstriction upon the foot CWI, even though the subject ratings of thermal sensation and pain do not support this notion.

Although the study was not designed to reveal seasonal changes, it spanned over 4 months, the latter two of which were winter months. Conspicuously, subjects investigated in January and February exhibited colder toes/feet during CWI and RW and a trend to colder fingers/hands during CWI. Whether these differences are attributable to the lower outdoor temperature in January and February or to other seasonal factors, e.g., physical activity level during the New Year’s holidays etc, remains to be investigated. Regardless, the seasonal differences in local temperature responses observed in the present study presumably had little impact on the main study conclusion that the thermal response of one limb is not transferable to the other limb.

The baseline autonomic and subjective responses of the participants did not differ between the two cold tests. Cortisol spillover has been reported ~15 min after a bilateral foot, but not after a unilateral hand, cold-pressor test (Larra et al. 2015). In view of this, it might be argued that the present 15-min interval between the tests was too short to allow full recovery. Nevertheless, any risk of confounding present overall results by any carryover effects from the first to the second CWI was minimized by the counterbalanced order of the trials.

In conclusion, the present findings demonstrate that the skin-temperature responses of the fingers/hand are not transferable to the toes/foot or vice versa, either during or after local cooling. Although the sensitivity of the cold-provocation test to predict cold injuries still needs to be determined, current results show that its use as a prognostic tool, if at all, must be limited to the tested limb.

## Abbreviations

CIVD: Cold-induced vasodilatation
CWI: Cold-water immersion
DAP: Diastolic arterial pressure
F-MT: Metatarsal region of the foot
H-MC: Metacarpal region of the hand
HR: Heart rate
*N*: Local skin-temperature wave in terms of >1 °C increase lasting for a minimum duration of 3 min
RW: Spontaneous (passive) rewarming phase
SAP: Systolic arterial pressure
*T*_avg_: Average temperature of each digit during every phase
*T*_max_: Maximum temperature
*T*_min_: Minimum temperature
*T*_onset_: Onset time of the first CIVD response
*T*_tympanic_: Tympanic temperature
WWI: Warm-water immersion
Δ*T*: Difference between the lowest temperature recorded just before the CIVD and the highest temperature reached during the CIVD
